# A new model of patient-reported outcome monitoring with a clinical feedback system in ostomy care: rationale, description and evaluation protocol

**DOI:** 10.1186/s12955-019-1261-3

**Published:** 2020-01-15

**Authors:** Kirsten Lerum Indrebø, Anny Aasprang, Torill Elin Olsen, John Roger Andersen

**Affiliations:** 1 Department of Surgery, Førde Central Hospital, Svanehaugvegen 1, 6812 Førde, Norway; 2 Western Norway University of applied Sciences, Førde, Norway; 3 Department of Surgery, Haukeland University Hospital, Bergen, Norway; 4 Centre of Health Research, Førde Hospital Trust, Førde, Norway

**Keywords:** Ostomy, Adjustment, Adaptation, Clinical feedback system, Routine outcome measure

## Abstract

**Background:**

Living with an ostomy can be challenging and adapting to life with an ostomy can be particularly complex, with regard to both the physical and psychosocial aspects. Follow-up with a stoma care nurse (SCN) is usually performed after surgery to support the adaptation process. In the present paper, we describe a new model of ostomy care, where a clinical feedback system (CFS) is implemented in order to improve the adaption process of patients with an ostomy. We also present a plan for evaluating patients experience with the CFS and their clinical outcomes.

**Methods:**

In this study, we include patients who had recently performed colostomy, ileostomy, or urostomy surgery. The intervention includes self-reported measures for adaptation to life with an ostomy and health-related quality of life (HRQoL), as well as patient experiences and satisfaction recorded by the clinical feedback system. The measures are electronically assessed before each clinical consultation at 3, 6, and 12 months after surgery. The scores are instantly analysed and graphically presented for use during the consultation and the patient and the SCN can discuss the findings. Patient experiences and satisfaction with care will be measured with the Generic Short Patient Experiences Questionnaire. Adaptation to the life with ostomy will be measured with the Ostomy Adjustment Scale, and HRQoL with the Short Form 36.

**Discussion:**

This study presents a novel approach that could lead to improved consultation, more patient involvement, and better adaptation to life with an ostomy.

**Trial register:**

ClinicalTrials.gov Registration Number: NCT03841071.

Date 18. February 2019 retrospectively registered.

## Background

Living with an ostomy defined as an opening constructed to extract bodily waste (flatus, faeces, or urine) can be challenging both physically and psychosocially. Patients with an ostomy lack a functional sphincter, and the waste empties involuntary in a pouch [1]. The importance of follow-up with a stoma care nurse (SCN) has been widely discussed, and novel approaches to tailor care more closely to the needs of these patients, particularly those struggling with ostomy-related adaptation and with a reduced health-related quality of life (HRQoL) could be useful. We here define ostomy-related adaptation as an individual’s degree of adaptive or maladaptive adjustment to specific important ostomy issues [2], while we define HRQoL as a multidimensional construct of the individual’s perception of physical, psychological, and social dimensions of health in a more broader and generic sense [3]. Ostomy care is in this project defined as the SCN’s follow-up of the patient in an outpatient clinic setting.

Although the major indications for an intestinal or urinary ostomy include malignancy in the urinary or gastrointestinal tract and indications for intestinal ostomies may include inflammatory bowel diseases, a wide range of diagnoses can lead to the need for an ostomy. Regardless of the need for ostomy surgery, the literature consistently describes negative feelings such as fear, insecurity, denial, shame, pain, dissatisfaction with appearance, and anger among patients after ostomy surgery [4]. These feelings could persist and result in a feeling of social stigma. Ostomy can involve changes in daily life, such as skin care, nutrition, clothing, work, leisure, social activities, sleeping, sexuality, and physical activity [4–8]. Two studies on short and long term adjustment found lowered adjustment in certain areas, such as sport and physical exercises, work, sexuality, body image, embarrassing accidents (such as leakage), and they would have done more things if they didn’t have had an ostomy [9, 10].

The HRQoL of ostomy patients is often negatively affected [11, 12], and may be lower than that in the general populations [13–17]; in fact, the HRQoL may remain low over several years after surgery [18]. The negative predictors for the adaptation process and HRQoL include a dependence on others for the management of the ostomy, leakage or fear of leakage, odour, noise and equipment failure, complications, and lack of social support and education [5, 18–20].

Lopez and Descerado et al. identified that dialog between the patient and the healthcare team was a major tool for a better adaptation to life with an ostomy [21]. Several studies specify that education programs, social support, and follow-up before and after surgery are imperative factors [7, 20, 22–26]. However, studies describing novel methods for consultations between the SCN and patients, and the manner in which patients provide feedback are lacking. There seems to be a gap in the literature when it comes to longitudinal studies describing how the patients adapt to life with ostomy.

A promising and relatively new clinical approach is the use of routine outcome monitoring (ROM) of the patient’s progress in treatment over time, using questionnaires that could include a clinical feedback system (CFS) in the consultation [27]. This approach has been successful in improving practice and outcomes within physical and mental-health care in a range of patient groups [28]. ROM/CFS can provide the clinician with detailed information that may have been overlooked or not previously regarded as important [29]. This gives clinicians’ better information on the state of the patient’s progress, and the clinician can share and discuss these results with the patient. Moreover, this knowledge can be used to evaluate, and if necessary, adjust treatment [30]. ROM/CFS is also valuable because it seems to have a positive effect on the communication and therapeutic relationship between the patient and the clinician [28, 31]. Web-based ROM/CFS appear to be a particularly feasible methodology in the healthcare setting, where it can be used to both monitor and evaluate treatment and as a research tool [32]. In the present paper, we describe a new model of patient reported outcomes (PRO) and CFS in ostomy care and a plan for evaluation.

### Aims

1. To study experiences and satisfaction with care in patients using PRO/CFS during the first 12 months after having an ostomy.
2. To study changes in the Ostomy Adjustment Scale (OAS) - profile and HRQoL in patients using PRO/CFS during the first 12 months after having an ostomy.
3. To study which items in the OAS score the patients using PRO/CFS rate as the most challenging during the first 12 months after having an ostomy.

## Methods

In this longitudinal study, we will include patients who have undergone urostomy, colostomy, or ileostomy operations, and who are included in the routine follow-up program of the outpatient ostomy clinic at the Department of Surgery, Førde Central Hospital from April 2018 to June 2021. The potential number of eligible new patients having ostomy pr. year is approximately 35. In this study, the ostomy patients undergo follow-up consultations at 3, 6, and 12 months after surgery. The inclusion criteria are (a) > 18 years of age; (b) living with a colostomy, ileostomy, or urostomy for up to 12 months; and (c) being able to talk, read and write Norwegian.

### Ethics and safety

The study conforms to the principles outlined in the Declaration of Helsinki. The Regional Committee of Ethics in Medicine, West-Norway, has approved the study protocols (registration numbers: 2016/255). Checkware, an agency which is used by the Western Norway Health Hospital Trust, has delivered the electronic version of the questionnaires. The study will use the highest security level possible in Norway to protect patient information. Each study participant will use his/her BankID with a code device or a cell phone, and a personal password. Questionnaires that are answered in the paper form, as well as the patient consent form, will be stored in a safe place in the Research Department.

### Power calculations

We base our power calculations on analysis of change in OAS over time. A priori power calculation should as be based on the smallest clinical meaningful difference over time. No minimally important effect size at a group level have been defined for OAS. Thus, we have to rely on research and consensus regarding this issue for PRO measures sin general. An effect size of 0.5 is a conservative estimate of clinical significance, and that effect sizes down to 0.3 may also be meaningful [33]. Consequently, our power calculation is based on a two-sided paired test (effect size = 0.4, correlation between measures of 0.3, 90% power, *p* ≤ 0.05) indicating that at least 68 paired observations would be required to detect reasonably robust 95% CI estimates of changes in the OAS. In order to take into account attrition we will aim to include 100 patients in the study.

### Intervention

SCNs educated in accordance with the requirements of the World Council of Enterostomal Therapists (WCET) conducts the systematic follow-up of ostomy patients in the outpatient clinic of Førde Central Hospital. The follow-up by SCN includes information, education in ostomy-related topics, skin and ostomy inspection and treatment, and to optimize the ostomy equipment as recommended in international and national guidelines and standards [34–36]. See Appendix 1 which describes the content in the previous and new follow-up consultations.

In the literature, SCN is also known as Wound, Ostomy and Continence Nurse or Enterostomal Therapist, but have been consistently regarded as SCN in this article.

The intervention in this study involves a ROM/CFS program where ostomy patients complete questionnaires before and after their consultation in the outpatient ostomy clinic approximately 3, 6, and 12 months after the surgery. The patient is expected to have the same SCN in all planned consultations.

### Procedure

When patients are invited to the 3-month follow-up visit in the outpatient department, they receive written information about the study and a participation consent form. Patients who want to participate in the study provide consent to their SCN. The SCN adds the questionnaires into the electronic database, and makes the questionnaires available to the patient.

On the day of the 3-month follow-up session, the participants can answer the questionnaires via a computer/Ipad at home before they arrive for the consultation or they can sit in a designated area in the waiting room for approximately 20 min prior to the consultation. During these 20 min, the patient can log onto the data system using his/her electronic identification to answer the questionnaires about sociodemographic data and clinical data, and complete the OAS and Short Form 36 (SF-36) questionnaires.

For patients without any personal electronic identification (BankID using a code device or a cell phone), the SCN allows the patients to access the questionnaires using a one-time code. In special cases, if the SCN logs on using his/her own code, the SCN will remain in the same room as the patient when he/she answers the questions for security reasons. Alternatively, the SCN asks the questions during the consultation and enter the answers into the database with the assistance of the patient, or we permit the patient to fill in a paper version of the questionnaire prior to admission for the consultation, and the SCN enters the answers into the database. In cases where the patient answers the questions via a computer or Ipad, the answers are available to the SCN before the patient arrives for the consultation.

During the consultation, the patient and SCN discuss the results and agree on the issues that may contribute to better adaptation and management of life with an ostomy. For patients who need help to answer the forms, the SCN discusses the answers with the patient.

The OAS items with the lowest scores will be presented first in the electronic report Low scores (1–3 on a Likert scale from 1 to 6) indicate that the patient is struggling with adaptation to the ostomy in the relevant life area, for example in work, leisure activities, travelling or body image and sexuality. The patient and SCN can then discuss strategies to achieve better adaptation. Similarly, the eight SF-36 domain scores are shown as bar charts, with different colours for each consultation. The patient and SCN can discuss methods for achieving adaptation in the items with low scores. For example, if the patient scores low on items relating to how safe he/she feels with regard to the ostomy bag, the SCN can discuss the type of bag used, the procedure, skin inspection and how to manage a situation with leakage from the equipment. Items that are scored better (4–6 on a Likert scale from 1 to 6) indicate better adaptation to the ostomy, and the SCN may choose not to focus on those during the discussion. Low SF-36 scores indicate reduced HRQoL, and the patient and SCN can discuss the reason for the low score and identify strategies to achieve a higher HRQoL.

The clinical part of the consultation, such as changing of the pouch, and observation of the ostomy and skin, is conducted before or after the discussion, depending on the preferences of the patient.

At the end of the consultation, the patient and SCN summarise the agreements and select the date and time for the next appointment.

After the consultation, the patients completes the Generic Short Patient Experiences Questionnaire scale (GS-PEQ) related to their experience during the consultation. This scale will be answered in a paper version.

The SCN fills in a set of clinical data after each consultation. The form has 2 parts, including a part about the diagnosis, type of ostomy, duration of ostomy, stoma site marking, treatment, and information given before surgery, and the patient’s degree of management of ostomy practical skills when he/she left the hospital after surgery. The SCN records these data after the first consultation. The second part of the form includes weight, description of the peristomal skin and the ostomy, complications, characteristics of body waste, any new diagnoses, and treatments initiated after surgery. The second part of the form is recorded after all consultations. This clinical data is also used in the discussion during the consultation and the findings will be available for future consultations.

### Implementation of the intervention

A pilot study was started with 15 patients, to evaluate, and if necessary, to adjust the intervention or other practical aspects of the study. In particular, we needed to test the functioning of the technology, and create a seamless process from the patient’s response to the graphical presentation of the questionnaire metrics during the consultation. As there was no need for technical or practical changes to the study plan based on that study, the patients in the pilot study are included in the actual study.

Two SCNs, including one of the study authors (KLI), will conduct the systematic follow-up of the patients. During the project, we will register the number of patients carrying their Bank ID, the number of patients requiring help from an SCN to fill in the scales, and the number of patients who needed to fill in a paper version of the questionnaires.

The project will be implemented within the usual follow-up schedule for the ostomy patients, and the findings will be documented in the hospital’s patient administrative system, as done previously. The project will not burden the patients in terms of money or a significant amount of additional time.

### Outcomes

#### GS-PEQ

To evaluate the patient’s experiences and satisfaction with ROM/CFS we will use the GS-PEQ. The Norwegian knowledge centre for health services questionnaires has developed and validated the 12–item scale, which contains questions about patient satisfaction and experiences with somatic outpatient services in Norway [37] (See Appendix 2).

#### OAS

To focus on the adaptation process, we will use a questionnaire without detailed evaluations of the ostomy equipment or clinical complications regarding the ostomy and skin, but with a focus on the consequences of body change and the adaptation to the new condition. Therefore, the Norwegian version of the OAS, originally developed by Olbrisch in 1983, will be used as measure of the primary outcome [2]. This 34-item scale developed by a psychiatrist, ostomy care nurses, patients, and students records a patient’s subjective adaptation to the physical, psychological, and social changes that occur after ostomy surgery. In particular, it records the patient’s employment status, marital relationship, social functioning status, self-image, and social life. The scale contains questions about the patient’s care of their ostomy, their opinions about the instructions they received about their ostomy, and their feelings about the SCN as well as the surgeon responsible for their ostomy surgery [2]. All the items are scored on a Likert scale from 1 (strongly agree) to 6 (strongly disagree); the total OAS scores can vary from 34 to 204, with a higher score indicating good adaptation to the ostomy [2]. The reliability of the OAS, as measured by Cronbach’s alpha, has been reported to be 0.87 [2], 0.89 [38], and 0.93 [9] in previous reports, and its test-retest correlation coefficients have been reported to be 0.72 [2] and 0.69 [9]. Previous studies also support the instrument’s construct validity [9, 39]. Mary Ellen Olbrisch, the researcher who designed the instrument, provided permission to use this scale for clinical research in Norway. In the electronic version, a graph shows the OAS sum scores from current and previous consultations. Furthermore, the patient’s most challenging metrics are reported first in the electronic report (see Appendix 2).

#### SF-36

The SF-36 is a well-validated, generic health scale that measures the outcomes that are known to be the most directly affected by disease and treatment [40]. The SF-36 is a self-reported questionnaire with eight subscales that measure physical functioning, body pain, role limitations due to physical health problems, role limitations due to emotional or personal problems, emotional well-being, social functioning, energy/fatigue, and perceived general health. Each subscale has a total score; the instrument can also be divided into two summary scores (based on factor analysis using oblique rotation): physical component scores (PCS; including domains such as physical function, physical role function, pain, and general health), and mental health component scores (MCS; including domains such as emotional role limitations, vitality, social function, and mental health). A single item that provides an indication of the perceived change in health is also included. The scores in each domain are converted to a scale from 0 to 100; higher scores are associated with better health-related quality of life (HRQoL). The scale has shown robust validity and reliability [41] (See Appendix 2).

### Statistical analysis

To assess the frequencies of the sociodemographic and clinical variables we will use descriptive statistics. Missing data on the questionnaires will be handled according to the procedures described for each questionnaire [2, 42]. In the analysis of patient experiences and satisfaction with care, descriptive results (number and percent) on each item of GS-PEQ at the 1 year follow up will be presented (aim 1). To study changes in OAS, PCS and MCS we will use longitudinal regression models with time as a (categorical) explanatory variable with an unstructured correlation matrix (aim 2). These models use data from all patients, even in patients with partially missing data. To study which items in the OAS score the patients range as the most challenging during first 12 months postoperative, mean item scores will be calculated and ranked (aim 3). SPSS software (version 25; IBM, Armonk, NY) will be used for all analyses.

### User involvement

A user panel including two patients with an ostomy has been involved in designing the study. Patients have co-produced the selection of questionnaires, and the workflow of the ROM/CFS according to patient’s needs. The user panel will also help interpreting findings in order to better disseminate aspects that are important from the patient’s point of view.

## Discussion

This study could be useful for improving the practice of follow-up by SCN for ostomy patients, as patients gives a more detailed description of his/her degree of adaptation to life with an ostomy. Furthermore, this may lead to more individualised consultations based on patients’ needs.

A strength of the current study is that the ROM/CFS concept has been implemented in other patient groups at Førde Health Trust. Thus, we have in-house resources available to implement and support this project both technically and clinically. The GS-PEQ scale is developed in Norway for use in evaluation of outpatient somatic healthcare [37]. The OAS, is well validated and in use worldwide [2, 38, 39, 43], and has been validated for use in the Norwegian population [9, 13].. The SF-36 questionnaire, is also widely used and well validated [41, 44, 45]. The sociodemographic and clinical forms are developed in cooperation with a reference group, including ostomy patients and SCNs with considerable experience in outpatient clinics. Feedback from both patients and SCNs not in the project can offer new perspectives and provide valuable feedback regarding the item’s relevance and ease of answering the questions.

This present study also has certain challenges. Based on our experience, we find that not all patients bring Bank ID equipment with them for the consultation, and may hence lack the necessary identification code to access the questionnaire. Therefore, we need to focus on minimising this occurrence during the project. Another challenge is the familiarity of patients in the use of IPad or computers, and their comfort with answering many questions (96 items) The lack of qualitative evaluation of the implementation is a methodological limitation. Our hospital has a small ostomy outpatient clinic with two SCNs (including the author), which may serve as a strength and limitation. For example, the close relationship between the author and the clinic can help with implementation, as the author can closely monitor the clinical part of the project; however, this close relationship also emphasises the need for a qualitative evaluation by external researchers on the new method of consultation. At present, the project does not have the financial resources to conduct qualitative studies, although this will be reconsidered if funding is secured.

## Data Availability

The dataset generated during this study will not be publicly available as the patient consent and approval from the Regional Committee for Medical and Health Research Ethics prevents sharing of individual patient level data in public repositories. However, the data will be available from the corresponding author upon reasonable request.

## Appendix 1

**Table 1:** Description of previous and the new follow-up consultations

Time	Previous follow-up consultations	New follow-up consultations
3 months postoperative	Semi structured data collection about the patient’s challenges and strengths. Dialog between patient and SCN. Education based on patient’s needs and what SCN think is necessary in accordance with guidelines and standards.	Data collection with use of validated electronically questionnaires at the clinic. The patient’s struggles are ranked first in the electronically report and areas that are functioning well are reported at the end of the scale. Dialog between patient and SCN. Education based on patient’s needs and what SCN think is necessary in accordance with guidelines and standards. The patient evaluates the consultation with use of GS-PEQ
6 and 12 months postoperative	Semi structured data collection about the patient’s challenges and strengths. Dialog between patient and SCN. Education based on patient’s questions and what SCN think is necessary in accordance with guidelines and standards.	Data collection with use of validated electronically questionnaires. The patient can answer the questionnaires from home some days before the consultation. The patient’s struggles are ranked first in the electronically report and areas that are functioning well are reported at the end of the scale. Results from previous consultations are shown. Dialog between patient and SCN. Education based on patient’s questions and what SCN think is necessary in accordance with guidelines and standards. Patient evaluates the consultation (CFS) with use of GS-PEQ

## Appendix 2

### Descriptions and links to the main questionnaires in English

1. **The Generic Short Patient Experiences Questionnaire (GS-PEQ).** HS-PEQ contains 12 questions about patient satisfaction and experiences with somatic outpatient services in Norway.
  Link to free full-text of the items: https://bmchealthservres.biomedcentral.com/articles/10.1186/1472-6963-11-88

2. **Ostomy Adjustment Scale (OAS).** OAS has 34 items on adjustment to life with an Ostomy.
  Link to free full-text of the items: https://www.o-wm.com/article/cross-sectional-study-determine-whether-adjustment-ostomy-can-predict-health-related-andor
  *Note: To see the items go to the results section of the paper, and click Table 2.*

3. **Short-Form-36:** The SF-36 is a self-reported questionnaire with eight subscales that measure physical functioning, body pain, role limitations due to physical health problems, role limitations due to emotional or personal problems, emotional well-being, social functioning, energy/fatigue, and perceived general health.
  Link to free full text of the questionnaire: https://www.rand.org/health-care/surveys_tools/mos/36-item-short-form/survey-instrument.html

## Abbreviations

CFS: Clinical Feedback System
GS-PEQ: Generic Short Patient Experiences Questionnaire
HRQoL: Health related quality of life
IC: Ileal conduit
OAS: Ostomy Adjustment Scale
ROM: Routine Outcome Monitoring
SCN: Stoma Care Nurse
SF-36: Short form 36
